# Autophagy and autophagy-related proteins in cancer

**DOI:** 10.1186/s12943-020-1138-4

**Published:** 2020-01-22

**Authors:** Xiaohua Li, Shikun He, Binyun Ma

**Affiliations:** 1 Henan Provincial People’s Hospital, Zhengzhou, 450003 China; 2 Henan Eye Hospital, Henan Eye Institute, Henan Key Laboratory of Ophthalmology and Visual Science, Zhengzhou, 450003 China; 3 People’s Hospital of Zhengzhou University, Zhengzhou, 450003 China; 4 People’s Hospital of Henan University, Zhengzhou, 450003 China; 5 Ophthalmology Optometry Centre, Peking University People’s Hospital, Beijing Key Laboratory of Diagnosis and Therapy of Retinal and Choroid Diseases, Beijing, 100044 China; 6 Department of Pathology and Ophthalmology, Keck School of Medicine of the University of Southern California, Los Angeles, CA 90033 USA; 7 Department of Molecular Microbiology and Immunology, Keck School of Medicine of the University of Southern California, Los Angeles, CA 90033 USA; 8 Department of Medicine/Hematology, Keck School of Medicine of the University of Southern California, Los Angeles, CA 90033 USA

**Keywords:** Autophagy, Autophagy-related proteins, Cancer suppressor, Cancer promotor, Cancer therapy

## Abstract

Autophagy, as a type II programmed cell death, plays crucial roles with autophagy-related (ATG) proteins in cancer. Up to now, the dual role of autophagy both in cancer progression and inhibition remains controversial, in which the numerous ATG proteins and their core complexes including ULK1/2 kinase core complex, autophagy-specific class III PI3K complex, ATG9A trafficking system, ATG12 and LC3 ubiquitin-like conjugation systems, give multiple activities of autophagy pathway and are involved in autophagy initiation, nucleation, elongation, maturation, fusion and degradation. Autophagy plays a dynamic tumor-suppressive or tumor-promoting role in different contexts and stages of cancer development. In the early tumorigenesis, autophagy, as a survival pathway and quality-control mechanism, prevents tumor initiation and suppresses cancer progression. Once the tumors progress to late stage and are established and subjected to the environmental stresses, autophagy, as a dynamic degradation and recycling system, contributes to the survival and growth of the established tumors and promotes aggressiveness of the cancers by facilitating metastasis. This indicates that regulation of autophagy can be used as effective interventional strategies for cancer therapy.

## Introduction

Fifty years ago, Christian de Duve, a Belgian scientist, firstly coined the term autophagy at the Ciba Foundation symposium on lysosomes in 1963 [1, 2], for which he shared the Nobel Prize in Physiology or Medicine in 1974 with Albert Claude and George E. Palade. There are three morphologically and mechanistically distinct types of autophagy in cells: macroautophagy, microautophagy and chaperone mediated autophagy [3], and usually macroautophagy is referred to as autophagy [4].

**Fig. 1:**
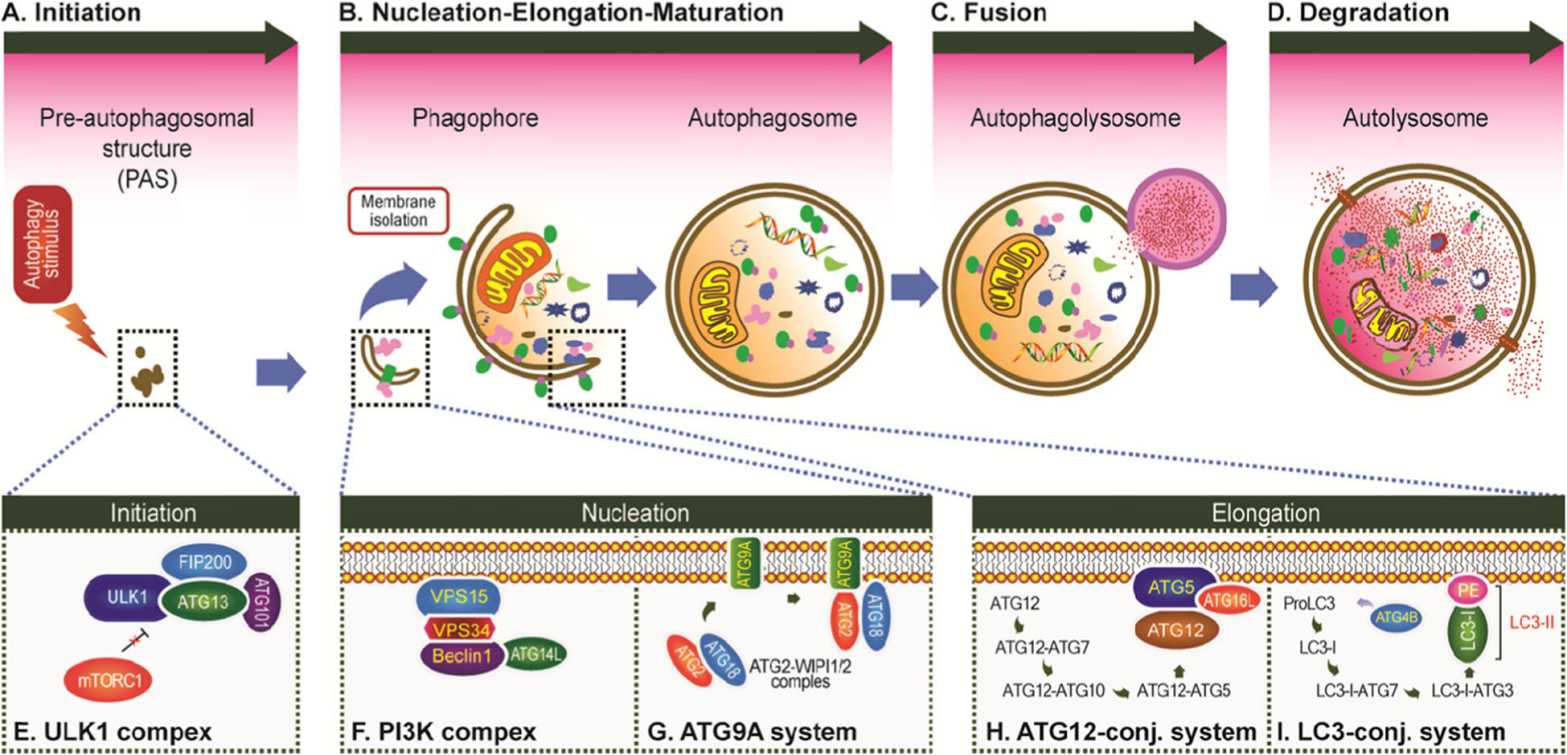
Schematic overview of autophagy. **a** Initiation, activation of ULK1 complex and multiple ATG proteins are engaged and localized to PAS. **b** Nucleation, ATG proteins and lipids are recruited to form phagophore; Elongation, cytoplasm and organelles are wrapped and engulfed during elongation of the phagophore; Maturation, completion and transport of the autophagosome. **c** Fusion, docking and fusion between autophagosome and lysosome. **d** Degradation, degradation of the cargos inside the autolysosome. **e** The ULK1 kinase core complex including ULK1, ATG13, FIP200, and ATG101. **f** The class III PI3K complex I including Beclin1, VPS34, VPS15, and ATG14L. **g** The ATG9A/ATG2-WIPI1/2 trafficking system including ATG9A, ATG2, and WIPI1/2. **h** The ATG12-conjugation system including ATG12, ATG7, ATG10, ATG5, and ATG16L. **i** The LC3-conjugation system including ProLC3, ATG4, LC3-I, ATG7, ATG3, and LC3-II (LC3-I/PE)

Autophagy is an intracellular evolutionarily conserved catabolic degradation process in which cytoplasmic macromolecules, aggregated proteins, damaged organelles or pathogen are delivered to lysosomes, and digested by lysosomal hydrolases to generate nucleotides, amino acids, fatty acids, sugars, and ATP, and ultimately recycled into the cytosol [5–13] (Fig. 1). This cellular self-digestion mediated by lysosome sustains, on the one hand, cell metabolism and survival during starvation and stress, and eliminates, on the other hand, damaged proteins and organelles to maintain protein and organelle quality and quantity [14, 15].

Although autophagy was found over 50 years ago, only within decade lots of studies elucidated the functions and roles of this ubiquitous process. Recent studies have indicated that autophagy plays a greater variety of pathophysiological roles in many disease processes, including cancer, neurodegeneration, autoimmune diseases, aging, cell death, heart disease and infection, and aids cell to clear damaged proteins, organelles, pathogens or aggregates, and has been proposed as a cell death mechanism, programmed cell death type II [16–21], whereas apoptosis is distinctively programmed cell death type I [22–24]. The potential ability of autophagy to modulate cell death makes it a therapeutic target in cancer [25, 26].

With its basic role in the turnover of proteins and organelles, autophagy has multiple physiological and pathophysiological functions. During tumorigenesis, autophagy plays an important role. In this review, the molecular basis of autophagy and its roles in cancer are summarized.

## Molecular basis of autophagy

Only a small amount of autophagy in cells is involved in maintaining homeostasis in physiological condition. When cells are stimulated by intracellular and extracellular factors e.g. starvation, hypoxia [27], some small molecular compounds [28], oxidation, and pathogen invasion [3, 29], a large number of autophagy is induced by the transduction of cellular signaling pathways, and many important autophagy-related proteins and their complex involved in the autophagic process [30].

### Process of autophagy

Physiologically, autophagy is an evolutionarily conserved, self-degradative, normal physiological process in cells, which is composed of several closely related steps including induction of autophagy, assembly and formation of autophagosome, autophagosome docking and fusion with lysosomal membranes, and degradation and recirculation of intra-autophagosomal contents in autophagolyosome [17, 31] (Fig. 1a-d).

#### Induction of autophagy

Induction of autophagy can be triggered by several intracellular and extracellular stimulus, e.g. nutrient starvation including depletion of total amino acids and serum starvation that strongly induces a high level of autophagy [27], oxidative stress that induces autophagy in order to recycle damaged organelles (e.g. mitochondria) and eliminate proteins aggregates [32], and inhibitors of TOR such as rapamycin and CCI-779 [17]. Under nutrient-rich condition, the active mTORC1 kinase hyperphosphorylates ATG13 and blocks the interaction of ATG13 with ULK1and FIP200. When cells are induced by those intracellular and extracellular stimulating factors, the ATG13 anchors ULK1 to a pre-autophagosomal structure (PAS), and then the almost all autophagy-related (Atg) proteins gather hierarchically onto the PAS (Fig. 1a), which is reported to be a crucial site of the cytoplasm to vacuole targeting (Cvt) and autophagosome formation [2, 33, 34].

As a dock structure for recruitment of ATG proteins, PAS plays a critical role during induction of autophagy [34, 35] Under autophagy-inducing conditions, the functional unit ULK1/Atg1 (including ULK1, ATG13, FIP200, and ATG101) acts as autophagy initiation complex, in which the ATG13 is a crucial protein for the PAS localization of ULK1 (Atg1 in yeast) and the interaction of FIP200 with ULK1, while the FIP200 (Atg11 and Atg17 in yeast) functions as a scaffold for downstream ATG protein assembly at the PAS. Once the ATG13 and ULK1 target to the PAS, all of these multiple ATG proteins are initially engaged and localized to the PAS, that is the initiation of autophagy [2, 33, 34] (Fig. 1a). Subsequently, the other functional units, including ULK1 complex, PI3K complex, ATG9A system, ATG12-conjugation system, and LC3-conjugation system, are targeted to the PAS in a hierarchical manner and involved in assembly and formation of autophagosome [12, 36–39].

#### Assembly and formation of autophagosome

Final formation of mature autophagosome includes nucleation of the multiple Atg proteins at PAS, elongation of the isolation membrane, and maturation of autophagosome, and four functional units are involved in these processes (Fig. 1b). The multiple Atg proteins gathering onto the PAS lead to the formation of a phagophore (or an isolation membrane) [40, 41]. The PAS is a potential nucleating site for forming the isolation membrane and recruits multiple Atg proteins. This nucleation process is initiated by the ULK1/Atg1 complex [42]. In response to nutrient starvation, the ULK1/Atg1 protein forms a complex with Atg13, FIP200/Atg17, Atg29, and Atg31, and this complex further associates with itself to generate the PAS scaffold complex, then the PI3K complex is gathered to the PAS and involved in forming phagophore through ATG14L interacting and binding to the ATG13 at PAS; and the ATG9A positive membrane vesicles associating with ATG2-WIPI complex (Atg2-Atg18 complex in yeast) are tethered to the PAS via interacting with the FIP200 (Atg17 and Atg11 in yeast). The multiple Atg proteins coordinate to generate the isolation membrane [42]. Once the first small ATG9A positive vesicles are fused at the PAS to form a phagophore, the bowl-shaped membrane is elongated continuously, and wraps and engulfs portions of cytoplasm and organelles. Finally, the isolation membrane, mediated by two ubiquitin-like ATG conjugation pathways, Atg12-Atg5 and Atg8/LC3 conjugation systems, forms a closed bilayer membrane structure, mature autophagosome with an inner and outer membrane [43] (Fig. 1b).

#### Autophagosome fusion with lysosomal membranes

Autophagosome docking and fusion with lysosomal membranes require the mature autophagosomes which will be transported to the perinuclear region for the autophagosome-lysosome fusion [44]. Autophagosomes can be formed randomly throughout the cytoplasm, whereas lysosomes are predominantly found in the perinuclear region. Therefore, once mature autophagosomes have been generated, they need to be delivered to the perinuclear region [45]. As long as autophagosomes arrive at the perinuclear region, they dock and fuse with lysosome immediately, and then form autophagolyosome (Fig. 1c).

#### Degradation and recirculation of autophagosomal contents

When autophagosome fuses with lysosomes to form autophagolyosome, many enzymes in lysosomes, e.g. lysosomal hydrolases, can degrade the inner membrane of the autophagosome and the cytoplasm-derived macromolecules, e.g. proteins and organelles, in the autophagosome into amino acids or peptides for reuse by cells (Fig. 1d).

### Autophagy-related proteins

Although autophagic structures by electron microscopy examination were firstly reported by Christian de Duve under 60 years ago, the molecular mechanism of autophagy regulation remained mostly unknown until discovery of yeast Atg genes in the 1990s, which greatly promoted the mechanistic understanding of autophagy and clarified the fact that autophagy plays important roles in various biological processes [46–49]. Functionally, multiple autophagy-related proteins regulate and control various stages of the autophagy formation, including initiation of autophagy, nucleation of the multiple Atg proteins at PAS, elongation of the isolation membrane, and maturation of autophagosome, trafficking of mature autophagosomes, autophagosome docking and fusion with lysosomal membranes, and degradation of intra-autophagosomal contents in autophagolyosome by a hierarchical manner [17, 31].

So far, more than 40 genes encoding Atg proteins have been identified in yeast [49], and most of the genes (e.g. Atg1-Atg10, Atg12-Atg14, Atg16-Atg18) are conserved between yeast and mammalian, which indicates that autophagy is an evolutionarily conserved process [50]. Klionsky et al. (2003) collectively named the genes encoding these proteins as ATG (AuTophaGy), which is used to represent the autophagy gene and its encoding protein [50] (Table 1; Fig. 1).

**Table 1:** Autophagy-related (Atg) genes and their protein function in autophagy

Genes		Protein function description	References
Mammals	Yeast		
ULK1/2 (Unc51-like kinase 1 and 2)	Atg1	Is part of the ULK-ATG13-ATG101-FIP200 complex and phosphorylates Beclin1; interacts with Atg13; is involved in initiation of autophagy, membrane targeting, membrane curvature sensing, and lipid vesicle tethering	[51–54]
ATG2A/B	Atg2	Is part of the ATG9/ATG12-WIPI complex, which is important for ATG9 recruitment to expand autophagosome	[55, 56]
ATG3	Atg3	E2-like enzyme in LC3 lipidation; autocatalyzes itself to form ATG12-ATG3 complex for maintaining mitochondrial homeostasis	[57–59]
ATG4A-D	Atg4	Cysteine protease to process Atg8 by removing its last amino acid; and deconjugate Atg8–PE; involved in LC3 activation and delipidation	[60, 61]
ATG5	Atg5	Is part of the ATG12-ATG5 complex involved in autophagosome formation/elongation, acting as an E3-like enzyme in LC3 lipidation; interacts with Atg16 and plays crucial roles in autophagy.	[62, 63]
Beclin1	Atg6	Is subunit of the VPS34-PI3K complex; recruits Atg14 or Vps38; interacts with Bcl-2; and lipid binding and membrane deformation	[64, 65]
ATG7	Atg7	E1-like enzyme interacting with E2 enzyme Atg10 or Atg3 involved in LC3 and ATG12 conjugation; and forms a thioester bond with Atg8	[66–68]
MAP 1 LC3A-C, GABARAPs, GATE-16	Atg8	Modifier; Ubiquitin-like module conjugated to PE and used as autophagosome marker; recognizes the cargo-specific adaptors; and in vitro membrane tethering	[69–71]
ATG9L1/L2	Atg9	Transmembrane protein; interacts with ATG2-WIPI complex; shuttles between PAS and peripheral organelles to deliver lipids/factors during phagophore expansion; and self-interaction	[72, 73]
ATG10	Atg10	E2-like enzyme in ATG12 conjugation with Atg5	[74–76]
ATG12	Atg12	Modifier; ubiquitin-like module conjugated to Atg5; forms an E3 complex with Atg5 and Atg16; and interacts with Atg3	[59, 62, 77]
ATG13	Atg13	Is part of the ULK-ATG13-ATG101-FIP200 complex involved in initiation of autophagy; targets mTOR signaling pathway; interact with Atg1 and bridges Atg1 and Atg17-Atg31-Atg29; recruits the Vps34 complex via Atg14; binds to LC3; and interacts with Atg101	[78–80]
ATG14L (Barkor)	Atg14	Is subunit of VPS34-PI3K complex; interacts with Beclin1 to assemble the autophagic-specific complex; membrane targeting and membrane curvature sensing; and promote membrane fusion	[81–83]
ATG16L1/L2	Atg16	Binds to ATG5-ATG12 complex acting as part of the E3 enzyme complex	[84–86]
RB1CC1/ FIP200	Atg17	Is part of the ULK-ATG13-ATG101-FIP200 complex involved in initiation of autophagy; interacts with Atg13 and Atg9; forms ternary complex with Atg31 and Atg29; and senses membrane curvature	[53, 78, 87]
WIPI1–4	Atg18	Is part of the ATG2-WIPI complex which is important for ATG9 recruitment to autophagosome; binds to PI3P; required for the retrograde transport of Atg9; and complexes with Atg2	[88, 89]
ATG101	–	Interact with Atg13 and forms the ULK-ATG13-ATG101-FIP200 complex	[90, 91]

-, This protein has not been identified

### Regulation and signaling of autophagy

In mammal cells, the starvation-induced autophagy is regulated by about 20 core ATG proteins, which can be classified into several functional units: (1) the ULK kinase core complex including ULK1/2, ATG13, RB1CC1/FIP200, and ATG101, (2) the autophagy-specific class III phosphatidylinositol 3-kinase (PI3K) complex including VPS34, VPS15, Beclin1, and ATG14L, (3) the ATG9A trafficking system including ATG9A, WIPI1/2, and ATG2A, (4) the ATG12 ubiquitin-like conjugation system including ATG12, ATG7, ATG10, ATG5, and ATG16L1, and (5) the LC3 ubiquitin-like conjugation system including LC3A/B/C, ATG7, ATG3, and ATG4A/B/C/D. These ATG proteins are recruited hierarchically proximal to the vacuole and organize the pre-autophagosomal structure (PAS) that is essential for autophagosome formation [12, 36–39] (Fig. 1e-i; Table 2).

**Table 2:** ATG proteins of mammals in the core machinery of autophagosome formation

Complex	Components	Roles of the proteins in the core machinery
The ULK kinase core complex	ULK1/2	Protein kinase and recruitment of ATG proteins to the PAS
	ATG13	ULK-binding protein and linker between ULK1/2 and FIP200
	RB1CC1/FIP200	Scaffold protein for ULK1/2 and ATG13
	ATG101	ATG13-binding protein
The class III PI3K complex I	VPS34	PtdIns 3-kinase catalytic subunit
	VPS15	Serine/Threonine protein kinase
	Beclin1	Component of PtdIns3K complex I and II
	ATG14L	Component of PtdIns3K complex I
The ATG9A/ATG2-WIPI1/2 trafficking system	ATG9A	Transmembrane protein required for autophagosome formation
	WIPI1/2	PtdIns3P-binding protein
	ATG2A	Interacts with WIPI1/2
The ATG12-conjugation system	ATG12	Ubiquitin-like protein conjugated to ATG5
	ATG7	E1-like enzyme
	ATG10	E2-like enzyme
	ATG5	Conjugated by ATG12
	ATG16L1	Interacts with ATG12 and ATG5
The LC3-conjugation system	LC3A-C, GABARAPs, GATE-16	Ubiquitin-like protein conjugated to PE
	ATG7	E1-like enzyme
	ATG3	E2-like enzyme
	ATG4A-D	LC3 carboxy-terminal protease, and deconjugating

#### ULK/Atg1 kinase core complex

During autophagy, autophagosome biogenesis commences at the PAS. In yeast, the Atg1 kinase core complex, consisting of the subunits Atg1, Atg13, Atg17, Atg29, and Atg31, is thought to play an essential and crucial role in the initiation of autophagy at the PAS, and has similar function to the ULK kinase core complex in mammal cells [92]. The ULK/Atg1 complex is mainly involved in receiving signals of cellular stimulation, recruiting ATG/Atg proteins to the PAS, organizing the vesicle cluster to form the phagophore, and governing elongation of the phagophore and formation of autophagosome [54, 93].

In human cells, the ULK1/2 is thought to serve similar and conserved functions as the yeast Atg1 [54]. In yeast, the core subunits of the Atg1 kinase complex are Atg1 and Atg13 [94]. When the cell is stimulated by starvation or other external stress, the target of rapamycin kinase complex (TOR) is inactivated, and then the Atg13 is dephosphorylated and binds greatly to Atg1 to form an activated Atg1-Atg13 dimer [95, 96]. The Atg17, Atg29, and Atg31 can assembly and form a trimeric complex Atg17-Atg31-Atg29 at the PAS [2, 97], and then serve as a preexisting scaffold for the recruitment of Atg1-Atg13 upon activation [98]. In mammal, the ULK1/2, a homologous protein of the kinase Atg1, forms a ULK1/2 kinase complex with ATG13 (homologous to Atg13 in yeast), FIP200 (homologous to Atg17 in yeast) and ATG101 (no homolog in yeast) [99] (Fig. 1e; Table 2).

Atg17 (FIP200) is the earliest protein to arrive at the PAS and adapts a highly elongated crescent shape [37, 93, 100]. The Atg17 is required specifically and associates physically with Atg1-Atg13 (ULK1/2-ATG13) complex, and the interaction between Atg17 and Atg1 is mediated by Atg13, indicating that Atge13 directly binds to both Atg1 and Atg17 and the Atg17-Atg13 complex formation plays an important role in normal autophagosome formation via binding to and activating the Atg1 kinase [33, 93, 96, 101, 102].

#### The class III PI3K complex I

In mammalian cells, the class III PI3K complex has two distinct types: complex I (PI3KC3-CI) and complex II (PI3KC3-CII). The both complexes share three core subunits: VPS34 (Vps34 in yeast), VPS15 (Vps15 in yeast), and Beclin1 (Atg6/Vps30 in yeast), and each complex contains a unique component: ATG14L/Barkor (Atg14 in yeast) of the autophagy-related complex I and Vps38 of the Vps-related complex II, which determines the localization of its own complex in the cell (Fig. 1f). The ATG14L (Atg14) can associate with and anchor the PI3KC3-CI to the PAS [103–106] and the Vps38 can localize the PI3KC3-CII to vacuolar and endosomal membranes [103, 107].

In the autophagy-specific PI3KC3-CI, VPS34, a catalytic PI(3) kinase, catalyzes phosphatidylinositol (PI) phosphorylation to form phosphatidylinositol 3-phosphate (PtdIns(3) P or PI3P). The PtdIns(3) P on autophagic membranes is essential for the elongation and completion of autophagosomes for it can bind and recruit the membrane-bound protein ATG18 to the bilayer membrane [108, 109].

In yeast, Atg6 mediates interaction with Atg14 that is crucial for localizing the PI3KC3-CI to PAS [110, 111]. The sole Class III PI3K, Vps34, is associated with the protein kinase Vps15, which functions as a Vps34 regulatory subunit [112]. Recently, it is reported that the interaction of Vps15-Vps34 with Atg14-Atg6 is mediated by Atg38, which was shown to play a crucial role in the complex integrity [113]. In mammalian cells, Beclin1 is a central regulator, which interacts with a multitude of proteins including ATG14L, UVRAG, Rubicon, and Bcl-2, etc. [114–117]. The Beclin1 has three functional domains including a N-terminal Bcl-2 homology 3 (BH3) domain, interacting with the Bcl-2 family protein Bcl-XL [118–120], a central coiled-coil domain (CCD), mediating interaction of Beclin1 with ATG14L and UVRAG [121], and a C-terminal evolutionarily conserved domain (ECD), mediating the interaction of Beclin1 with VPS34 and activation of VPS34 kinase activity to regulate the size and number of autophagosomes [110, 111, 116, 122] (Fig. 1f; Table 2).

#### The ATG9A/Atg9 trafficking system

After the ULK/Atg1 complex is formed, the next step is recruitment of ATG9A/Atg9-containing cytoplasmic vesicles (ATG9A/Atg9 vesicles), which is a crucial step of autophagosome formation and plays an essential role in the nucleation step of autophagosome formation in eukaryotes (from yeast to mammals) [73, 123, 124]. The ATG9A/Atg9 can be phosphorylated by ULK/Atg1, and then the phosphorylated ATG9A/Atg9 is required for the recruitment of LC3/Atg8 and WIPI1/2/Atg18 to the site of autophagosome formation and the expansion and elongation of phagophore [125].

Mammalian ATG9A, the yeast Atg9 homolog, is the sole multi-spanning transmembrane protein within the core machinery of autophagosome formation and has 6 highly conserved transmembrane helices and 2 cytosolic NH2- and COOH-terminal domains that are involved in interactions with other ATG components in both yeast and mammals [126–128].

The ATG9A/Atg9 self-interacts and self-associates within membranes into a higher-order assembly [129]. Recent studies indicated that the majority of Atg9 in the yeast are incorporated on small cytoplasmic vesicles with diameters of 30–60 nm, namely Atg9 vesicles [123, 130]. It is estimated that 3 Atg9 vesicles contain approximately 30 molecules of Atg9 each assemble at the PAS [123, 131], and subsequently the Atg9 is integrated into the outer autophagosomal membrane. Once the autophagosomes fuse with vacuole, the Atg9 are recycled as new Atg9 vesicles [102, 123, 132]. The level of Atg9 expressed in cells correlates with the frequency of autophagosome formation and the number of autophagosome [133]. The Atg9 vesicles are originated and transported from the Golgi apparatus [73, 102, 123, 127]. In normal physiological conditions, Atg9 localizes to and cycles between the trans-Golgi network (TGN) and early and late post-Golgi endosomes [134, 135]. A recent study suggests that autophagosome formation occurs where ATG9 vesicles coalesce with the ER [136].

In yeast, Atg9 recycling from PAS is regulated by the Atg2-Atg18 complex [37, 102]. The Atg18 and Atg2 are peripheral membrane proteins. The Atg18 as a downstream effector of class III PI (3) K localizes to PAS via binding to PtdIns(3) P [137]. Recent reports showed that the Atg18-Atg2 complex may play an important role in transporting the membrane structures during autophagosome formation through binding to Atg9 and form an Atg9·Atg2-Atg18 complex on the surface of the PAS and further regulate cycling of Atg9 [102, 138, 139].

In mammals, the WIPI (WD-repeat protein interacting with phosphoinositides) proteins, including WIPI1, WIPI2, WIPI3, and WIPI4, have a similar function to the Atg18 [88, 140, 141]. The WIPI1/2-ATG2 complex is involved in forming ATG9A·WIPI1/2-ATG2 trafficking system, mediating and regulating cycling of ATG9A, and promoting formation of LC3-positive autophagosomes in autophagy [140]. The WIPI1/2-ATG2 (Atg18-Atg2 in yeast) complex localizes to the expanding edge of the isolation membrane and plays a key role in the elongation and/or closure of the isolation membrane [43, 142] (Fig. 1g; Table 2).

#### The ATG12/Atg12-conjugation system

During autophagosome formation, two ubiquitin-like conjugation systems are required including the ATG12/Atg12 and LC3/Atg8 conjugation systems, and as many as eight ATG proteins are involved in both conjugation systems, which contribute to and are tightly associated with expansion of autophagosomal membrane.

In yeast, the Atg12, a ubiquitin-like protein, is covalently linked to its substrate Atg5 and forms an irreversible Atg12~Atg5 conjugate [38]. The Atg12-conjugation system is similar to the E1-E2-E3 activation and ligase present in the ubiquitination pathway, in which Atg12 is activated by Atg7, an E1-like enzyme [143], and then is transferred to Atg10, an E2-like enzyme [144], and is finally conjugated to its substrate protein Atg5 [38]. The Atg12~Atg5 conjugation has no typical E3 enzyme. The Atg5 of the Atg12~Atg5 conjugate further interacts with a small coiled-coil protein, Atg16, to form a ~ 350-kDa Atg12~Atg5-Atg16 complex [145, 146].

In mammals, the ATG12, activating by the E1 enzyme ATG7, is conjugated to ATG5 via the E2 enzyme Atg10 and then the ATG12-ATG5 conjugate can be stabilized by ATG16L proteins and further form ATG12-ATG5- ATG16L complex of approximately 800 kDa, which is important for the formation of the LC3 conjugation system [58, 62, 147] (Fig. 1h; Table 2).

#### The LC3/Atg8 -conjugation system

The LC3/Atg8-conjugation system is located downstream of the ATG12/Atg12 system in the context of Atg protein organization.

In yeast, the Atg8, another ubiquitin-like protein in yeast, is covalently linked to phosphatidylethanolamine (PE) after its C-terminal Arg117 residue is removed by a cysteine protease, Atg4, to expose to Gly116 [148]. The Atg8 is activated by the E1-like enzyme Atg7 [143], and then transferred to the E2-like enzyme Atg3 [149], and eventually the Atg3 conjugates Atg8 with the PE through an amide bond [39]. The Atg8-PE conjugate can be cleaved by Atg4 to release free Atg8, indicating that the Atg8-PE is reversible [148]. Most of Atg8 exist in the unconjugated form under normal conditions, but when autophagy is induced by starvation, most of Atg8 are activated, transferred, and converted to the PE-conjugated form [150].

In mammalian cells, there are several homologues of yeast Atg8 including LC3, GATE16, GABARAP and ATG8L. The LC3 has been best investigated and characterized as an autophagosome marker in mammalian cells [151–155], which forms an Atg8-like conjugation system, called the LC3-conjugation system (Fig. 1i; Table 2).

LC3, microtubule-associated protein light chain 3, is a soluble protein with a molecular mass of approximately 17 kDa. The LC3 is firstly synthesized as a precursor protein (proLC3) [156], then the C-terminal peptide of the proLC3 precursor is cleaved by mammalian ATG4B homologues to form LC3-I with an exposed C-terminal glycine [151, 157, 158]. Catalyzed by mammalian ATG7 and ATG3 homologues, cytosolic LC3-I is then activated by the E1 enzyme ATG7 and transferred to the E2 enzyme ATG3, and finally is modified to a membrane-bound protein, LC3-II, by conjugating to the amino group of the lipid phosphatidylethanolamine (PE) (LC3-I/PE). Then, the Atg12-Atg5-Atg16 complex acts as an E3 enzyme for the conjugation reaction of LC3-II (LC3-I/PE) [154, 155, 157] (Fig. 1i), which corresponds to the Atg8-PE-conjugated form in yeast [151, 159]. The ATG4B has been reported that it is the sole enzyme to efficiently cleave LC3 precursors and LC3-I/PE among four human homologues of yeast Atg4 (Atg4A-D) [151]. Thus, the both ubiquitin-like systems are intimately involved in formation of PAS, assembly and formation of autophagosome, and subsequent biogenesis of autophagy.

## Autophagy in cancer

Physiologically, autophagy, by eliminating damaged proteins and organelles during stress and aging, plays critical roles in regulating organismal development, cooperating with the adaptive immune system, sustaining energy homeostasis and maintaining protein and organelle quality control [11, 160–164].

In diseases, such as neurodegenerative diseases [165, 166], infectious diseases [11, 167, 168], and metabolic diseases [14], dysfunctional autophagy leads to the accumulation of abnormal and damaged proteins and organelles and formation of intracellular aggregates, and then prevents the ability of autophagy to battle and eliminate infectious pathogens [11, 161, 167, 169].

In cancer, autophagy can play neutral, tumor-suppressive, or tumor-promoting roles in different contexts and stages of cancer development [25, 170–173], which is determined by nutrient availability, microenvironment stress, pathogenic conditions, and the presence of an immune system.

### Dual role of autophagy in cancer

**Fig. 2:**
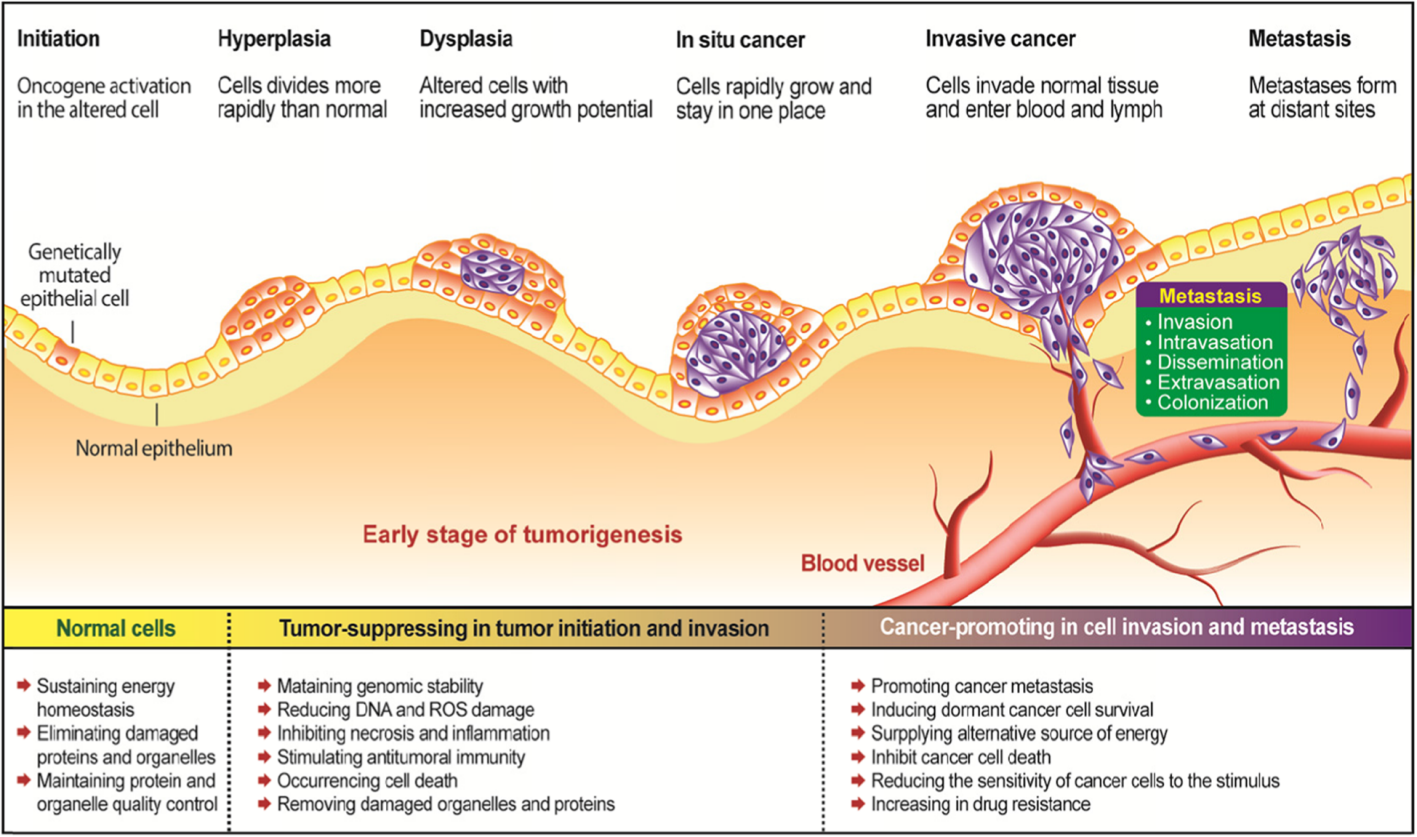
Dual role of autophagy in tumorigenesis. Tumorigenesis begins with an oncogene mutation in the epithelial cell that makes the cell more likely to divide. The genetically altered or abnormal cells and its descendants grow and divide uncontrolled and rapidly at Hyperplasia stage. At Dysplasia stage, the overgrowing cells change their original form and behavior, have increased growth potential, and consist of more immature cells than mature. In situ cancer, the cells grow rapidly, but do not go into the process of maturation, have lost their tissue identity, and grow without regulation. In the malignant tumor (invasive cancer), the overgrowing cells invade neighboring areas and blood circulation systems from the primary tumor site by rupturing basal membrane. Metastases occur when cancer cells reach to the distant parts through lymphatic system and blood circulation. Autophagy plays dual roles during tumorigenesis including tumor-suppressing role during the early stage and cancer promoting role during the late stage

In cancer development, autophagy plays a dual role depending on type, stage or genetic context of the cancers [174–179]. On the one hand, via its protein and organelle quality control function, autophagy can maintain genome stability, prevent chronic tissue damage, cell injury, and inflammation, and inhibit accumulation of oncogenic p62 protein aggregates, and then prevent tumor initiation, proliferation, invasion, and metastasis, thereby function as a tumor suppressive mechanism, especially in the early stage of tumorigenesis [180–182]. Autophagy is important for the quality control of the cells such as removing damaged mitochondria, and its defective proteins (e.g., heterozygous knockdown Beclin1 and Atg7 in mice) promote the malignant transformation and spontaneous tumors [183–185]; on the other hand, once the tumors progress to late stage, autophagy can function as a cellular protective, survival, and defense mechanism, maintain functional mitochondria, reduce DNA damage, and enhance the survival and resistance of the cancer cells against stress (e.g., nutrient deprivation, hypoxia, DNA damage metabolic stress, and chemotherapy), and then sustain tumor metabolism, growth, and survival and then mediate tumor promotion and development, finally promotes tumorigenesis and causes resistance to therapeutic agents [180, 182, 186]. It is reported that autophagy can contribute to the aggressiveness of the cancers by facilitating metastasis [187–189] (Fig. 2). The effect of autophagy on cancers is dependent on multiple factors including tumor microenvironment, cancer type and stage, and genetic background.

### Autophagy suppresses tumorigenesis

Autophagy, the lysosome-mediated cellular self-digestion, acts as a cellular quality-control mechanism to sustain cell metabolism and its protein and organelle quality control during starvation, eliminates damaged proteins and organelles that accumulate during stress, and suppress chronic tissue damage, then prevent tumor initiation, especially in the early stage of tumorigenesis [11, 180]. Several indirect evidences indicate that autophagy acts as a tumor suppressor (Fig. 2).

#### Defective autophagy contributes to tumorigenesis

Through the identification of Beclin1, an essential autophagy gene, autophagy is first linked to human cancer. The Beclin1, as a haploid-insufficient tumor suppressor, is mono-allelically deleted in human hepatocellular carcinoma (HCC), breast, ovarian, and prostate cancers [114, 115, 190, 191] and in mice tumor prone [192]. It is reported that the expression of Beclin1 in cancer tissues was down-regulated in 44 patients with hepatocellular carcinoma, and it was concluded that autophagy might inhibit tumorigenesis [190]. The spontaneous frequency of malignancies is higher in the Beclin1^+/−^ mouse model [192, 193], indicating that autophagy is a tumor-suppression mechanism [11, 25, 190, 194].

A number of studies on the ATG genes relevance to human cancers showed that other ATG genes are also oncogenically associated, including ATG2B, ATG5, ATG9B, ATG12 and ATG16L1. The frameshift mutations with mononucleotide repeats have been found in ATG2B, ATG5, ATG9B and ATG12 genes in gastric cancer and colorectal cancer, which may be involved in cancer development by deregulating the autophagy process [195]. The homozygote deletion of ATG5 predisposed to liver tumors with high penetrance mouse model [196]; the somatic point mutations of ATG5 are also identified in 135 patient samples of gastric cancer, colorectal cancer, and hepatocellular carcinoma [197]. The compartment-specific expression of ATG16L1 in epithelial cancer cells inhibited tumor growth [198].

Taken together, whether the expression of the intact autophagy genes is downregulated in cancers or the spontaneous frequency of cancer malignancies is increased due to autophagy-related gene deficiency, indicating that the intact autophagy functions as a cancer suppression mechanism by limiting genome damage and mutation and constraining tumor initiation.

#### Autophagy inhibits necrosis and inflammation

Autophagy is a central regulator of the inflammasome, and the chronic inflammation is a common future of early cancer development [199–202]. The oncogene activation can cause neoplasia and inflammation, and the inflammatory conditions can increase cancer risk. The autophagy-deficient tumors display an increased level of necrosis and inflammation, indicating that the intact autophagy can inhibit neoplasia, inflammation and cancer [203, 204]. Defective autophagy lead to tissue damage, necrosis, chronic inflammation, and genetic instability, which can increase the incidence of cancer by altering the tumor microenvironment, elevating oxidative stress and creating cancer-causing mutations [204, 205]. In autophagy-defective cells and tissues, the failure to eliminate damaged proteins and organelles leads cellular dysfunction and death, and then stimulates an inflammation condition, and creates ultimately a cancer-prone environment [206].

Among mammal ATG proteins, Beclin-1, ATG5, ATG7, ATG12, ATG16L1 and LC3B are the most studied with respect to inflammation [207], and defects in autophagy are linked to many inflammatory diseases [208, 209] and cancer [210].

#### Accumulation of p62/SQSTM1 promotes tumorigenesis

The p62, also called sequestosome 1 (SQSTM1) in humans, a multifunctional adaptor protein, is a selective substrate of autophagy. In intact autophagy, the p62/SQSTM1 possesses a short LC3 interaction region (LIR) that facilitates direct interaction with LC3 and causes p62 to be specifically degraded by autophagy, while defective autophagy is a mechanism for p62 upregulation commonly observed in human tumors, so the level of p62 has been used as a marker for inhibition of autophagy or defects in autophagic degradation [211–213].

The aberrant accumulation of p62 has been detected in the cases of gastrointestinal cancer [214], prostate cancer [215, 216], hepatocellular carcinoma [217–219], breast cancer [220, 221], lung adenocarcinoma [222], suggesting that p62 accumulation correlates with cancer progression and autophagy suppresses tumorigenesis by limiting p62 accumulation [213, 223, 224].

### Autophagy mediates cancer promotion

Once the tumors progress to late stage, autophagy can promote the survival and growth of the established tumors by removing toxic oxygen radicals or damaged proteins, maintaining mitochondrial function, sustaining metabolism and survival in stress, and preventing diversion of tumor progression to benign oncocytomas [180–182]. Many investigations have shown that autophagy is the major contributor for cancer cells substantially survival [225–227]. It is reported that autophagy can contribute to the aggressiveness of the cancers by facilitating metastasis [187–189]. Moreover, autophagy as a cellular defense mechanism may reduce the effect of treatments of most chemotherapeutic agents (Fig. 2).

#### Autophagy prevents cancer cell damage

Autophagy is robustly activated in cancer cells under a multitude of stress conditions, including starvation, growth factor deprivation, hypoxia, damaging stimuli and proteasome inhibition, so elevated levels of autophagy have been observed in many tumor types, e.g. the essential autophagy gene Beclin1 was upregulated in colorectal cancer, gastric cancer, liver cancer, breast cancer, and cervical cancer [228–231], suggesting that the enhancement of autophagy can promote tumorigenesis and overexpression of the Beclin1 plays a crucial role in tumor formation.

Autophagy functioning as a cancer promotion mechanism is mainly based on its role involved in removing damaged mitochondria, inhibiting DNA damage, maintaining genome stability, limiting inflammation, and finally preventing cancer cell damage under the conditions of stress [14, 213, 232]. Normal mitochondrial function, e.g. mitochondrial respiration, is required for tumorigenesis [233], t the accumulation of morphologically abnormal mitochondria and mitochondrial dysfunction have been found in the autophagy-defective tumors [196, 234–236], indicating that intact autophagy can remove damaged mitochondria and contribute to tumorigenesis. The activation of the DNA damage response, gene amplification, DNA copy number variations and an elevated mutation rate has been found in the autophagy-deficient cancer cells [237]. Autophagy prevents genome damage and promotes tumor cell survival in a model of mammary cancer [194]. Autophagy is induced in hypoxic tumor regions and is required for tumor cell survival and for limiting inflammation [226]. (preventing cancer cell damage). All of these evidences indicate that the survival function of autophagy can be commandeered by tumors to prevent cell damage and promote tumorigenesis under conditions of metabolic stress.

#### Autophagy promotes cancer metastasis

During cancer progression, metastasis is an extremely complex process that indicates a more advanced stage and a poorer prognosis and accounts for most cancer-related deaths [238]. The metastasis of primary tumor can be divided into a series of stages including invasion of tumor cells from the primary tumor site, intravasation and survival in blood circulation systems, dissemination of the malignant cancer cells through the circulation systems to reach a capillary bed and adhere to the vessel walls, extravasation of the cancer cells at a distant site, and finally colonization of disseminated tumor cells at their destination organs [239–242].

Autophagy plays a complex and stage-specific role and promotes multiple steps during cancer metastasis [243]. During the early stage of metastasis, the autophagy may act as a suppressor of metastasis by preventing tumor necrosis and restricting inflammatory cell infiltration [243]; on the other hand, in the advanced stages of metastasis, the autophagy may act as a promoter of metastasis by promoting dissemination of the malignant cancer cells in the circulation [244], enhancing colonization of detached metastatic cell in the destination organs [245], and inducing metastatic cells to enter dormancy and survive in the new environment [246].

Autophagy is upregulated during cancer metastasis. Once the metastatic cancer cells successfully establish distant colonies in their destination organs, autophagy begins to play a critical role and the autophagic flux is induced to respond various environmental stress including hypoxia, nutrient deprivation and detachment from the ECM [187, 240, 247, 248]. Using the autophagy marker, LC3B, various studies have identified an association between increased autophagy and metastasis in several types of cancer including breast cancer metastasis [249, 250], melanoma metastases [251], hepatocellular carcinoma [189], and glioblastoma [252]. These evidences indicate that autophagy promotes cancer metastasis and enhances the aggressiveness of cancer cells [253].

#### Autophagy inhibit cancer therapy

Cancer cells have common characteristics including increased metabolic demands, high level cellular proliferation, evading growth suppressors, resisting cell death, enabling replicative immortality, inducing angiogenesis, activated invasion and metastasis, and enhanced cellular stress, which require autophagy to be activated to maintain energy, enhance stress tolerance, limit damage, and prevent death in these cells.

Autophagy plays a cytoprotective or pro-survival role in cancer cells and can be induced by most cancer treatments including radiation therapy [254–256], chemotherapy [257, 258], histone deacetylase inhibitors in colon cancer cells [259], arsenic trioxide (As2O3) in malignant glioma cells [260, 261], Temozolomide (TMZ) in malignant glioma cells [262], γ-irradiation in breast cancer, prostate cancer, colon cancer and malignant glioma [263–265], resveratrol in ovarian cancer [266], TNFα in breast cancer cells [267], IFNγ in Hepatocellular carcinoma (HCC) [268], imatinib lung carcinoma cell [269], rapamycin in malignant glioma cells [270], and tamoxifen in breast cancer and Glioblastoma [271, 272], and the autophagy, in turn, functions as a cellular defense and protection mechanism to prevent cancer cell death upon treatment, enable a state of dormancy in residual cancer cells post treatment, contribute to cancer recurrence and metastasis, and inhibit cancer therapy and tumor cell killing [246, 273].

Given the pro-survival role, the inhibition of the autophagy has been shown to enhance and increase the efficacy of anticancer therapy, implying that autophagy inhibition is a potential valuable approach in combination with other anticancer therapeutic approaches to enhance cancer treatment [181, 182].

## Conclusions and perspectives

Autophagy, as a cell survival pathway, plays an important role in cancer, and can help to prevent bioenergetic failure by metabolic stress and maintain protein and organelle quality and quantity, and contributes to all aspects of tumorigenesis, including tumor initiation, progression and development, and maintenance of the malignant state. Cancer cells divide relentlessly, and they are also metabolically stressed. As cancer cells grow, spread, and form solid tumors or flood the blood with abnormal cells, they always face an acute problem increasing hypoxia and nutrient deprivation, which may promote their death and prevent their growth, progression, and development, and autophagy is essential for surviving these stresses and maintaining tumorigenesis. Autophagy also plays key roles in controlling the tumor microenvironment, in suppressing tumor during the early stage and promoting cancer during the late stage, and in the therapeutic response.

Autophagy has a dual role both in progression and inhibition of cancer. Hitherto many data support a dynamic role of autophagy in cancer, both as a tumor suppressor early in progression and as a cancer promotor later in tumor maintenance and therapeutic resistance. In the early tumorigenesis, autophagy, as a survival pathway and quality-control mechanism, contributes to normal cell physiology metabolism and provides biological materials and energy in response to stress, and as a dynamic degradation and quality-control mechanism, eliminates damaged proteins and organelles and prevents tumor initiation. Once the tumors progress to late stage and are established and subjected to the environmental stresses including limited angiogenesis, nutrient deprivation, and hypoxia, autophagy, as a dynamic degradation and recycling system, contributes to the survival and growth of the established tumors and promotes aggressiveness of the cancers by facilitating metastasis.

Regulation of autophagy can be used as effective interventional strategies for cancer prevention and therapy by preventing cancer development, limiting tumor progression, and increasing the efficiency of cancer treatment. On the one hand, autophagy, as one type of programmed cell death, is ubiquitous in various cancer, functions as a tumor suppressor pathway, facilitates the degradation of oncogenic molecules, and finally prevents development of cancers. So defective or inadequate levels of autophagy can lead to cancer. Investigations showed that all chemotherapeutic agents and radiotherapies induce cancer metabolic stress and concomitant inhibition of autophagy, indicating that the autophagy regulation represents a significant direction in the development of anticancer therapies. On the other hand, autophagy, the type II programmed cell death, is involved in several signaling pathways during tumorigenesis via coordinating with apoptosis, the type I programmed cell death. Under stress conditions such as hypoxic or low-nutrition environments autophagy facilitates the survival of tumor cells, and at same time, apoptosis prevents the survival of cancer cells, indicating that autophagy and apoptosis, as two catabolic pathways, are essential for organismal homeostasis and tumor microenvironment. Investigations have now shown that autophagy and apoptosis are interconnected and coordinated by several molecular nodes of crosstalk, such as interaction of Beclin1 with Bcl-2, UVRAG with Bif-1, and ATG12 with the Mcl-1, etc.

So far, some standard cancer treatments have saved, or at least prolonged, many lives. However, the most severe clinical issue is the frequent tumors progression and cancer recurrence after treatment, mainly due to therapeutic resistance. It can be sure that autophagy can facilitate the tumor cells survival and deal with anticancer therapy. Therefore, in the near future, standard cancer treatment combining with regulation of autophagy activity, promoting or preventing by autophagy inducers or inhibitors based on tumorigenesis and cancer stages, can be considered as a potential anticancer therapy. However, further investigations should be done to understand and clarify how autophagy contributes to the development and treatment of cancer, how the autophagy pathway can be targeted and regulated, and how the activity of autophagy pathway can be monitored and quantified during cancer prevention and therapy.

## Data Availability

Data sharing not applicable to this article as no datasets were generated or analyzed during the current study.

## Abbreviations

As_2_O_3_: Arsenic Trioxide
ATG: autophagy-related proteins, such as ATG1, ATG4, ATG5 ATG7 etc.
BH3: Bcl-2 homology 3 domain
CCD: Coiled-coil domain
Cvt: Cytoplasm to vacuole targeting
ECD: Evolutionarily conserved domain
ECM: Extracellular matrix
ER: Endoplasmic reticulum
FIP200: FAK-family interacting protein of 200 kDa
GABARAP: γ-aminobutyric-acid-type-A-receptor-associated protein
GATE16: Golgi-associated ATPase enhancer of 16 kDa
HCC: Hepatocellular carcinoma
LC3: Microtubule-associated protein 1A/1B-light chain 3
LC3-I: The cytosolic form of LC3
LC3-II: The conjugate form of LC3-I with phosphatidylethanolamine (PE) (LC3-I/PE)
LIR: LC3 interaction region
p62/SQSTM1: a ubiquitin-binding protein p62, sequestosome 1
PAS: Pre-autophagosomal structure
PI: Phosphatidylinositol
PI3KC3-CI and PI3KC3-CII: Class III phosphatidylinositol 3-kinase complex I and II
ProLC3: The precursor protein form of LC3
PtdIns(3)P: Phosphatidylinositol 3-phosphate (PI3P)
RB1CC1: RB1-inducible coiled-coil protein 1
TGN: Trans-Golgi network
TMZ: Temozolomide
TOR: Target of rapamycin
ULK1/2: Unc51-like kinase 1 and 2
UVRAG: UV radiation resistance-associated gene
VPS15: Vacuolar protein sorting 15
VPS34: Vacuolar protein sorting 34
WIPI1/2: WD-repeat protein interacting with phosphoinositides proteins 1 and 2
